# PD76 Cost-Effectiveness Analysis Of An Influenza Vaccination Program In Algeria, Egypt, Saudi Arabia, Turkey, and the United Arab Emirates

**DOI:** 10.1017/S0266462324003313

**Published:** 2025-01-07

**Authors:** Arun Jones, Suzanna Mongan, Ahmed Abdou Saleh, Khelafi Rachida, Majdy Idrees, Mohamed Awad Tag El Din, Önder Ergönül, Kyoo Kim, Nesrine Aouida, Alona Kondratenko, Dr Hansoo Kim

## Abstract

**Introduction:**

Influenza causes seasonal epidemics and is a significant burden. Annual vaccination is an effective way to prevent influenza and its complications. However, vaccination coverage remains low in North Africa and the Middle East. Quadrivalent vaccines offer protection but require monetary investments. This study aimed to quantify the costs and benefits of subunit quadrivalent influenza vaccination among healthy adults in the selected countries.

**Methods:**

A decision tree was used to model the costs and outcomes of treatment with an inactivated subunit quadrivalent vaccine, compared with no vaccine, in healthy adults from the societal and payer perspectives. Outcomes measured include productive days gained through reduced absenteeism and the proportion of the population acquiring influenza. Each country’s expected health resource utilization costs for influenza were determined through literature searches and consultations with clinical experts. Deterministic and probabilistic sensitivity analyses were undertaken to characterize the effects of parameter uncertainty on our results.

**Results:**

We found that in every country the proportion of people who acquired influenza was 2.4 times lower in vaccinated individuals than in the unvaccinated. This resulted in between 0.9 and 1.5 more productive days worked for vaccinated individuals. The average cost savings from the societal perspective ranged from USD12.36 to USD276.85 per person. Deterministic sensitivity analysis indicated that influenza prevalence was the strongest driver of the model results. A probabilistic sensitivity analysis of over 1,000 simulations resulted in cost savings 54.4 to 99.3 percent of the time.

**Conclusions:**

Vaccination with a subunit quadrivalent influenza vaccine results in cost savings when compared with no vaccination in every country studied. We therefore conclude that, in terms of cost effectiveness, vaccination with a subunit quadrivalent influenza vaccine is dominant over no vaccination in Algeria, Egypt, Saudi Arabia, Turkey, and the United Arab Emirates.

